# Experimental and computation assessment of thermomechanical effects during auxetic foam fabrication

**DOI:** 10.1038/s41598-020-75298-w

**Published:** 2020-10-27

**Authors:** Richard Critchley, Victoria Smy, Ilaria Corni, Julian A. Wharton, Frank C. Walsh, Robert J. K. Wood, Keith R. Stokes

**Affiliations:** 1 Survivability and Advanced Materials, Centre for Defence Engineering, Cranfield University, Defence Academy of the United Kingdom, Shrivenham, SN6 8LA UK; 2 National Centre of Advanced Tribology at Southampton (nCATS), Mechanical Engineering, Faculty of Engineering and Physical Sciences, University of Southampton, Highfield, Southampton, SO17 1BJ UK; 3 Applied Cognitive Science Group, Centre for Electronic Warfare, Information and Cyber, Cranfield University, Defence Academy of the United Kingdom, Shrivenham, SN6 8LA UK; 4 nC2, Enterprise Consultancy Unit, University of Southampton, Southampton, SO17 1BJ UK

**Keywords:** Engineering, Mechanical properties, Polymers

## Abstract

Auxetic foams continue to interest researchers owing to their unique and enhanced properties. Existing studies attest to the importance of fabrication mechanisms and parameters. However, disparity in thermo-mechanical parameters has left much debate as to which factors dominate fabrication output quality. This paper provides experimental, computational, and statistical insights into the mechanisms that enable auxetic foams to be produced, using key parameters reported within the literature: porosity; heating time; and volumetric compression ratio. To advance the considerations on manufacturing parameter dominance, both study design and scale have been optimised to enable statistical inferences to be drawn. Whilst being unusual for a manufacturing domain, such additional analysis provides more conclusive evidence of auxetic properties and highlights the supremacy of volumetric compression ratio in predicting Poisson’s ratio outcomes in the manufacture process. Furthermore statistical results are exploited to formulate key recommendations for those wishing to maximise/optimise auxetic foam production.

## Introduction

Auxetic materials are a class of material that is of particular interest due to unique and enhanced mechanical properties^1^. Unlike traditional materials, auxetics are characterized by a counterintuitive behaviour; when a tensile load is applied in one direction they expand in another direction^2,3^. More simply, they become fatter, laterally, when stretched. A wide range of auxetic materials, such as polymers, metals, ceramics, composites, laminates, and fibres have been manufactured, but are yet to find large scale commercial use^4–7^. This is partly due to the difficulty in making reliable and predictable auxetic materials^8^. While manufacturing methods such as additive manufacturing^9–11^ have been used to overcome these repeatability issues, auxetic foams manufactured through the conventional thermo-mechanical approach first reported by Lakes^12^ remain of interest owing to their low cost, availability and ease of application^2^.

To date, the thermo-mechanical auxetic process has been extensively applied^3,12–26^ and has typically used open-cell polyurethane foam^12–17,27–35^. Across many of these studies disparities in experimental thermo-mechanical parameters are often reported but each study successfully produces auxetic foams. This has led to much debate regarding which are the key factors influencing the process. Wang et al.^24^ state that the main physical parameters influencing the auxetic transformation process is the volumetric compression ratio (VCR), the processing temperature and the heating time. However, Bianchi et al.^16,17,20^ consider only VCR as the main parameter responsible for a successful conversion, with the other parameters having a secondary influence. Additional parameters reported to influence auxetic foam fabrication include the composition of the material, its relative density, cell size, cell shape, and humidity^16,17,19–21,24^.

This paper provides new insights into the mechanisms that enable auxetic polyurethane foams to be produced over a selected range of experimental thermo-mechanical parameters reported in the literature. The parameters of porosity, heating time and VCR are experimentally and computational explored, while a fixed heating temperature representative of the upper repeated value reported in literature is used to understand the thermal transfer throughout the foams during the thermo-mechanical process. Particular focus is placed on the generation of a dataset affording statistical analysis. Such an approach provides supplementary evidence from which more definitive conclusions can be established on parameter dominance, allowing discrimination amongst the parameters most effective at producing auxetic outputs. To the authors’ knowledge, no such statistical approach has been reported to date for auxectic form fabrication.

## Experimental methodology

### Sample production

**Figure 1:**
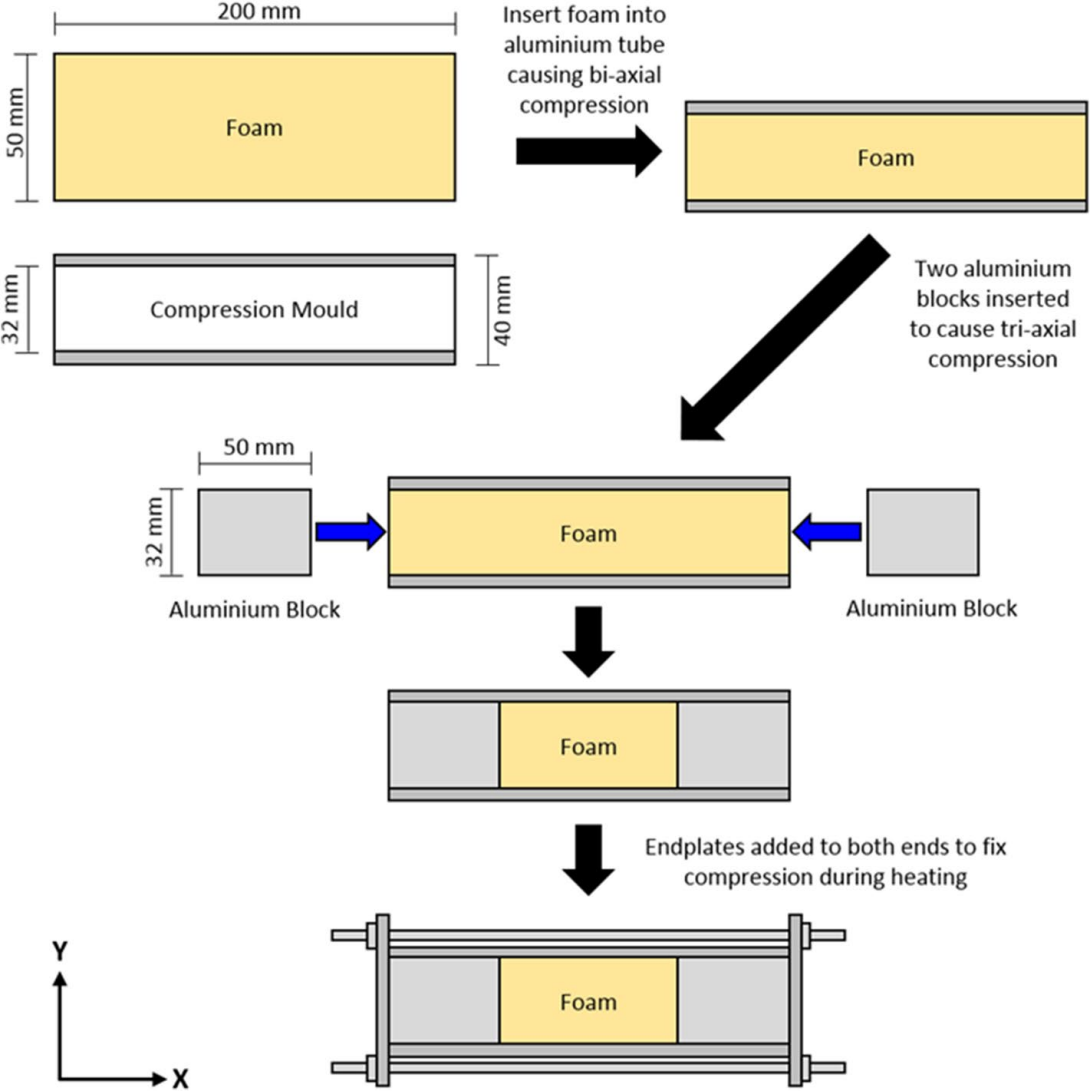
Auxetic foam fabrication process. Pore rise direction is in the *y*-axis.

Commercially available black 45 and 10 pores per inch (PPI) polyurethane foam, of densities 27 and 34 kg m^−3^ was supplied by Recticel, UK as sheets of dimensions 1500 mm × 2000 mm × 50 mm. The foams were cut into three sample sizes: (1) 50 mm × 50 mm × 200 mm, (2) 50 mm × 50 mm × 160 mm and (3) 50 mm × 50 mm × 120 mm, and inserted into aluminium moulds (internal dimensions 32 mm × 32 mm × 200 mm). The foam samples were subjected to tri-axial compression resulting in a target VCR of 4.88 (200 mm length), 3.91 (160 mm length) and 2.93 (120 mm length), which were selected to allow comparison with literature^3,14,19,20,24,36–39^ (see Fig. 1).

Constrained samples were heated at 200 °C in a fan assisted oven for different fabrication times (ranging between 25, 30, 35, 40, 45, 50, 55 and 60 min). The fabrication temperature used represented the upper repeatable value reported in the open literature and exceeded the glass transition temperature of polyurethane (114 °C^20^). To determine the softening temperature for the supplied PU foams, Differential Scanning Calorimetry (DSC) has been carried out using a Mettler-Toledo instrument, model DSC821e, where the heating programme set up used the STARe SW version 9.01^40^ software to vary a temperature between 25 and 550 °C, at a scan rate of 10 °C min^−1^.

For each combination of VCRs and foam types, a K-Type thermocouple (RS Components, UK) was located at centre of the samples to monitor the internal temperature during fabrication to determine the time taken for heat to diffuse to the centre. Temperature data was captured over a 60 min period (at 1 Hz sampling rate). Three replicates for each VCR foam combination were undertaken to determine an average response.

After heating, the fabrication moulds were removed from the oven and allowed to cool for a minimum of 2 h until both sample and mould reached ambient room temperature (typically 21–23 °C). The foam samples were removed from moulds and hand stretched along the *x*-axis direction to relax the sample. Samples were weighed once using a Sartorius Talent, TE2145S electronic balance (± 0.0001 g), while the *x-*, *y-*, and *z*-dimensions were measured three times (± 0.5 mm) and averaged to calculate sample density immediately after fabrication. A total of 192 foam samples was fabricated using this methodology giving 48 fabrication combinations (four samples per combination), to provide a suitable dataset for both mechanical testing and statistical analysis.

### Mechanical testing

Foam samples were bonded to two steel endplates of dimensions 35 mm × 40 mm × 2.5 mm, using a two-part epoxy resin which was cured at room temperature for 24 h. Samples were loaded along the *x*-axis, up to strain of 1.0 at 0.1 intervals at a rate of 32.5 mm min^−1^ using a Hounsfield A10 tensometer in a laboratory of ambient room temperature (typically 21–23 °C). Relative humidity was not measured in this study. If any evidence of failure was observed in either the sample or the force-extension response the test was immediately stopped. For each interval of strain, sample displacement was temporally halted, and the change in sample length (${\text{d}}\varepsilon_{{{\text{length}}}}$) and width (${\text{d}}\varepsilon_{{{\text{width}}}}$) was measured (± 0.5 mm). Poisson’s ratio (ν) can be expressed using the following equation:

1 $$ {\upnu } = - \frac{{{\text{d}}\varepsilon_{{{\text{width}}}} }}{{{\text{d}}\varepsilon_{{{\text{length}}}} }} $$

### Computed tomographic analysis

The compression effect on the foam structure was investigated using an X-Tek benchtop computed tomography (CT) system (with a maximum scan area: 200 mm × 200 mm, with a 5 µm resolution). To replicate the volumetric compression within the CT scanner, replica moulds and endplates were manufactured from Perspex to enhance the contrast between the mould and the foam. A single replica of each foam sample used in the thermo-mechanical process (“Sample production” section) was inserted into the mould and tri-axially compressed.

To optimise the number of scans, data collection and scan time, symmetry about the mould’s centre was assumed, resulting in a region of interest (30 mm × 30 mm × 50 mm) being imaged using an X-ray source of 110 kV with a voxel resolution of 0.0433 mm. The scan data was reconstructed using CTPro version 2.2^41^ via filtered back projection algorithm before being cropped, concatenated and converted to 8-bit grayscale using ImageJ version 1.49o software^42^. From this point forward each scan volume will be referred to as a sub-volume due to its larger volume.

**Figure 2:**
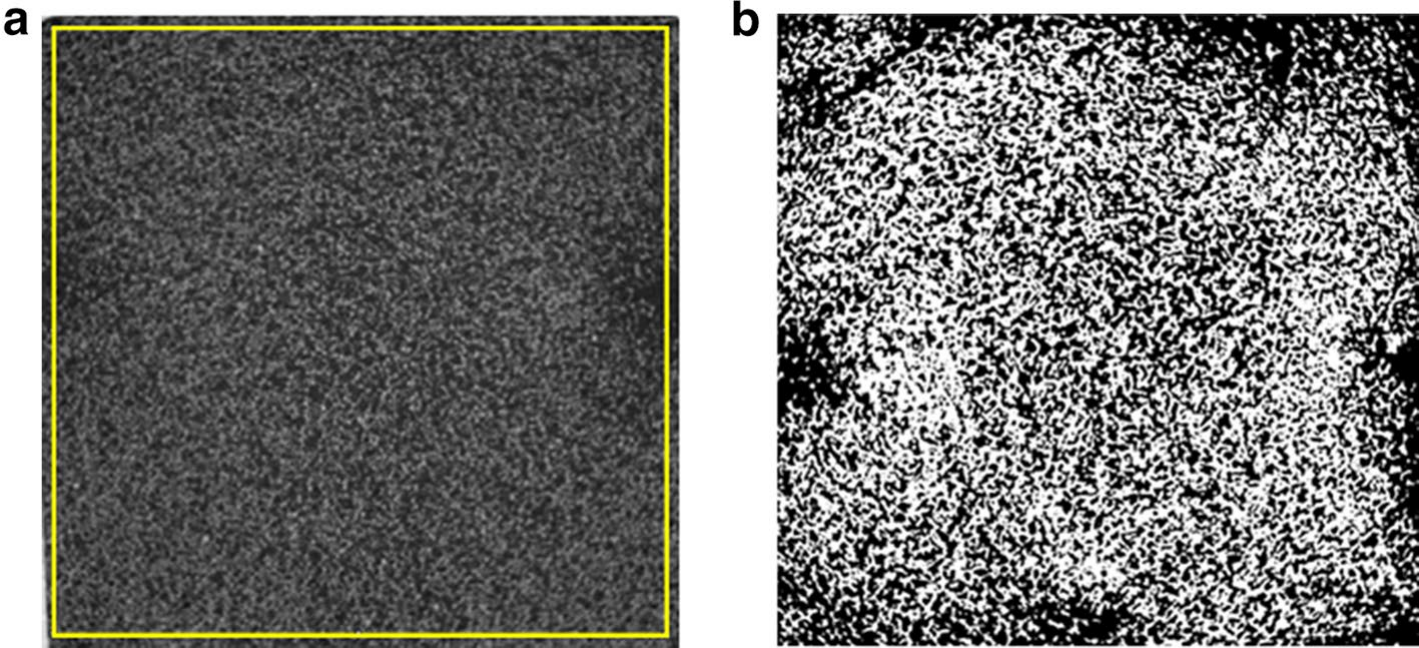
Example of 45 PPI foam structure along the *x*–*y* axis captured using CT where: (**a**) pre- and (**b**) post-processed data. Yellow outline indicates area of crop extraction to ensure no artefacts from the Perspex mould.

Foam relative density (ρ*/ρ_s_), where ρ* is the cellular material density and ρ_s_ is the fabrication material density^43^, was calculated by binarising the 8-bit volumes (i.e., image digitisation into either zeros or ones) using the threshold standard presented by Reinhart^44^ to extract the foam structure. A region was cropped to include the internal region of the foam and exclude artefacts that were in the upper and lower 50 voxels of the scan. The relative density was calculated by dividing the number of voxels representing the foam structure by the total number of voxels within a given slice using the standard voxel counter included within ImageJ to represent relative density as a function of distance in the *z*-axis. An example of the pre- and post-processed CT data is shown in Fig. 2.

### Heat model

**Figure 3:**
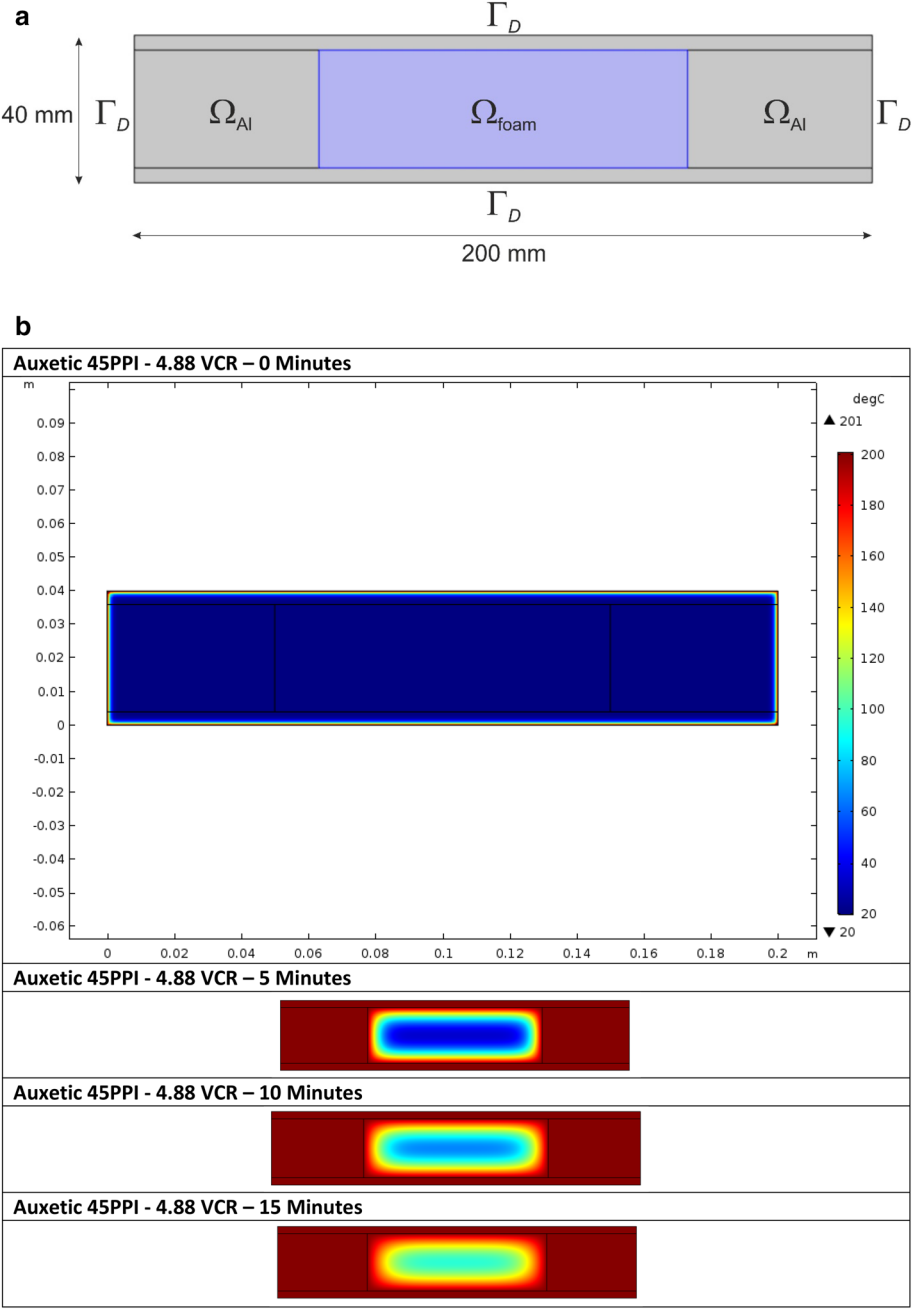

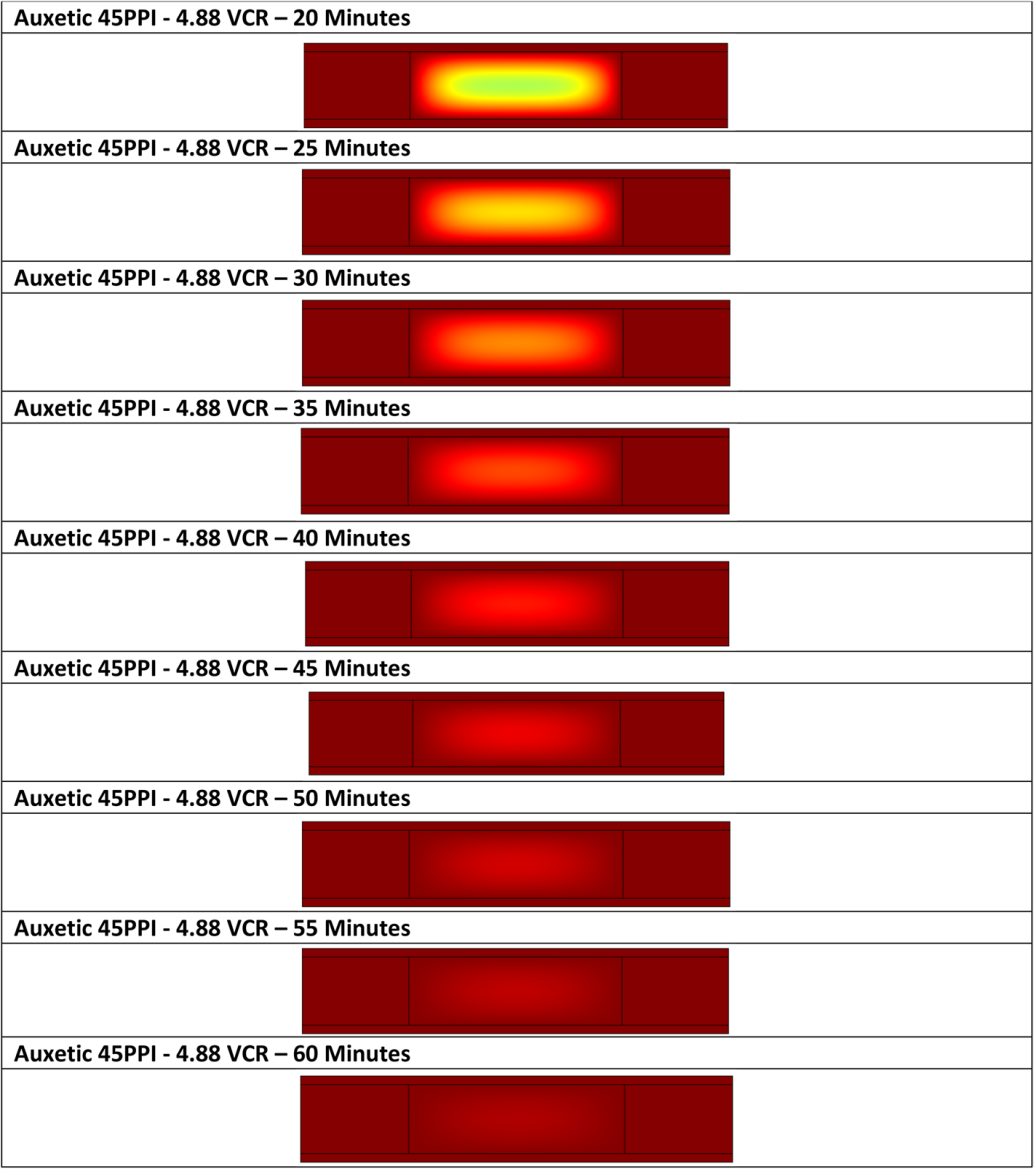
(**a**) Schematic of the two-dimensional model used for the heat transfer calculation highlighting geometry domains and Dirichlet boundary conditions and (**b**) COMSOL model heating profiles at 0 to 60 min indicating localised temperature in the *x*–*y* axis.

A two-dimensional heat transfer model was developed using COMSOL Multiphysics Version 5.3a^45^ software to analyse the heat transfer within the foam. The model comprised of two geometry domains (${\Omega }_{{{\text{foam}}}}$ and ${\Omega }_{{{\text{Al}}}}$) each with their own properties (Table 1). The mould components were defined as a solid material and foam as porous. It should be noted that when a material is defined as porous in COMSOL, unlike real foams, which comprised of a randomly distributed foam cell network, it is suspected that COMSOL homogenises the porous media into a single medium^46^. A fixed heating source of 200 °C was applied to the external boundaries (Dirichlet boundary condition, ${\Gamma }_{{\text{D}}}$) of the mould (Fig. 3a). The governing equation for this problem is the steady-state heat equation for conduction^47^.

**Table 1:** Heat model parameters for the three volumetric compression ratios; values were sourced from the CES Edupack software^49^.

Foam properties			
Volumetric compression	4.88	3.91	2.93
**45 PPI**			
Density (kg m^−3^)	159	130	92
Thermal conductivity (W m^−1^ K^−1^)	0.025	0.025	0.025
Specific heat capacity (J kg^−1^ K^−1^)	1650	1650	1650
Ratio specific heat	1.4	1.4	1.4
**10 PPI**			
Density (kg m^−3^)	203	156	113
Thermal conductivity (W m^−1^ K^−1^)	0.025	0.025	0.025
Specific heat capacity (J kg^−1^ K^−1^)	1650	1650	1650
Ratio specific heat	1.4	1.4	1.4
**Mould properties**			
Material	(7020 aluminium alloy)		
Density (kg m^−3^)	2810		
Thermal conductivity (W m^−1^ K^−1^)	143		
Specific heat capacity (J kg^−1^ K^−1^)	911

The model was meshed using a quad element of size 1 mm and simulated between 0 to 3600 s, at 300 s intervals, yielding a mean solve time of 10 to 15 s. The model calculated temperature variations with respect to time, using the heat equation^48^. An example of the spatial temperature with respect to time is given in (Fig. 3b).

### Statistic methodology

Two statistical approaches were utilised and undertaken using SPSS version 26^50^. Firstly, multiple regression enabling the statistical assessment of how variation in the output dependent measure (i.e., Poisson’s ratio) is attributable to variation in the independent variables, i.e., the fabrication parameters^51,52^. Inspection of partial correlation coefficients can be used to determine the unique contribution of each independent variable in predicting the dependent variable. Secondly, discriminant function analysis is presented. Here the categorisation of production outcomes as either successful or unsuccessful in producing auxetic foam samples enables: (1) the ranking of fabrication parameter dominance in successfully producing auxetic foams, and (2) the likelihood of certain parameter combinations in producing auxetic foams^53^.

## Results

### Poisson’s ratio

**Figure 4:**
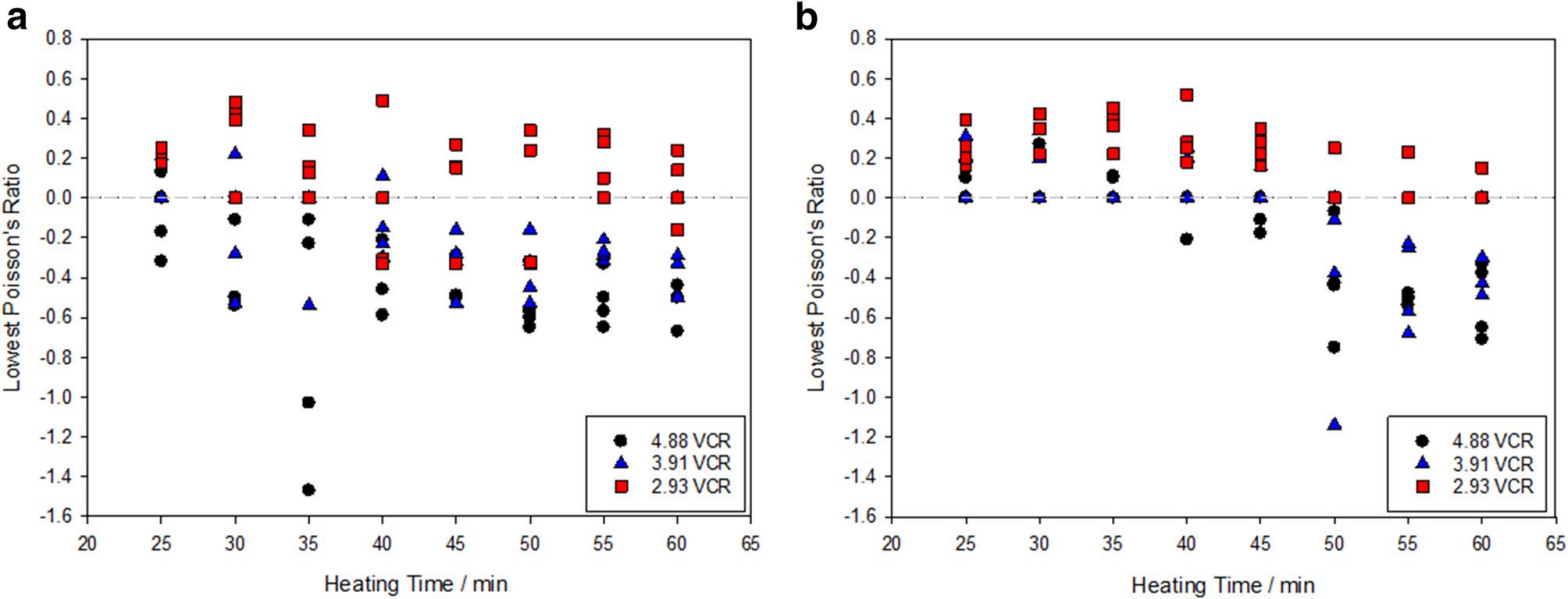
Lowest exhibited Poisson’s ratio for each polyurethane foam sample produced with respect to heating time for: (**a**) 45 PPI and (**b**) 10 PPI samples. VCR: 2.93 (filled square), 3.91 (filled triangle) and 4.88 (filled circle).

**Figure 5:**
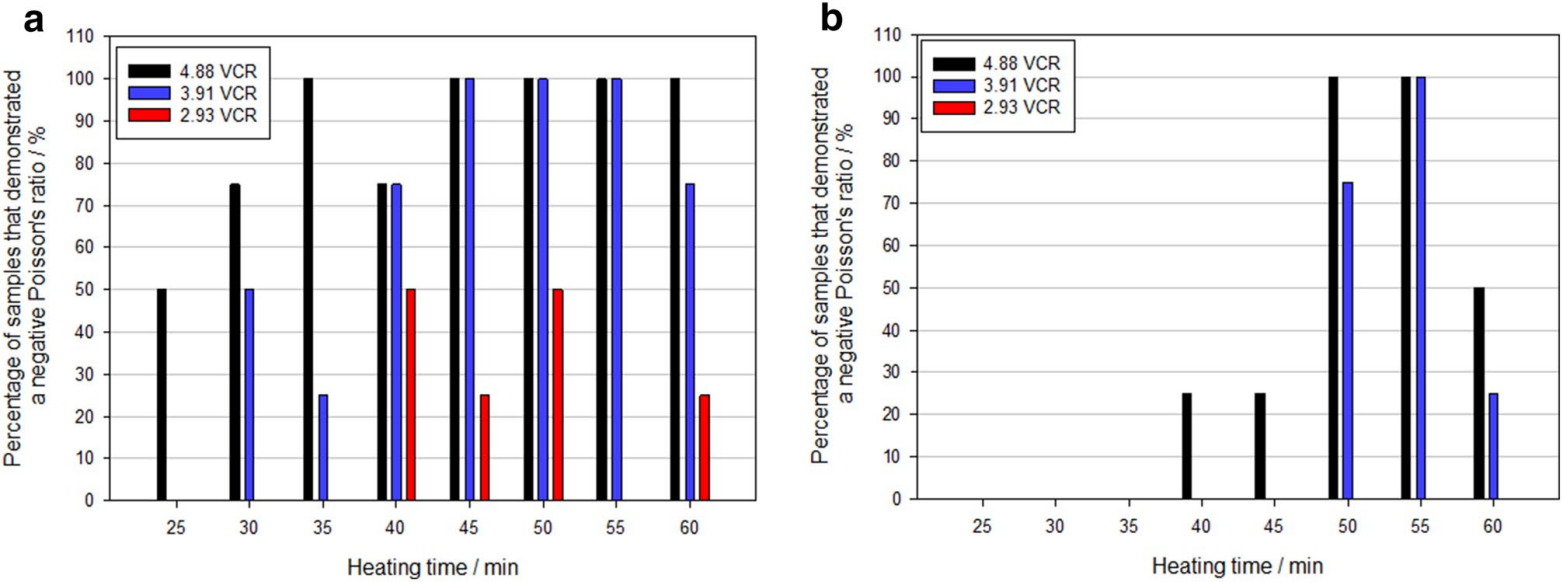
Percentage of foam samples that demonstrated a negative Poisson’s ratio for a given heating time and volumetric compression ratios of 4.88 (black bar), 3.91 (blue bar) and 2.93 (red bar) for: (**a**) 45 PPI and (**b**) 10 PPI polyurethane foams.

The success rate of producing polyurethane foams with a negative Poisson’s ratio are shown in Figs. 4 and 5. In total, 42% of the samples demonstrated a negative Poisson’s ratio, across the range of heating times, volumetric compression ratios and porosities. The 45 PPI foams were found to be more successful at auxetic conversion (57% conversion rate) compared to the 10 PPI foams (21% conversion rate). Typically, samples exhibited the lowest negative Poisson’s ratios (NPRs) between − 0.10 to − 0.67 and − 0.07 to − 0.75 for the 45 PPI and 10 PPI foams, at strains of 0.1 to 0.3 respectively. However, in rare instances foams exhibited NPRs greater than − 1, indicating anisotropic behaviour, similar to those reported in^26,34,54^.

The higher heating times were more effective in producing auxetic behaviour. As heating times decreased there was evidence of a transition period in which the fabrication repeatability decreases, resulting in a failure to produce an NPR. The onset of this transition period appears to be related to the VCR, with higher VCRs, taking longer for the uncertainty period to develop. When the transition period was exceeded, auxetic behaviour was no longer evident. In some instances, no transition period was evident as no heating time produced repeatable auxetic behaviour. To understand this behaviour VCR and heating time were individually explored, before statistically analysed to ascertain their influence on the auxetic fabrication process.

### Heating time

**Figure 6:**
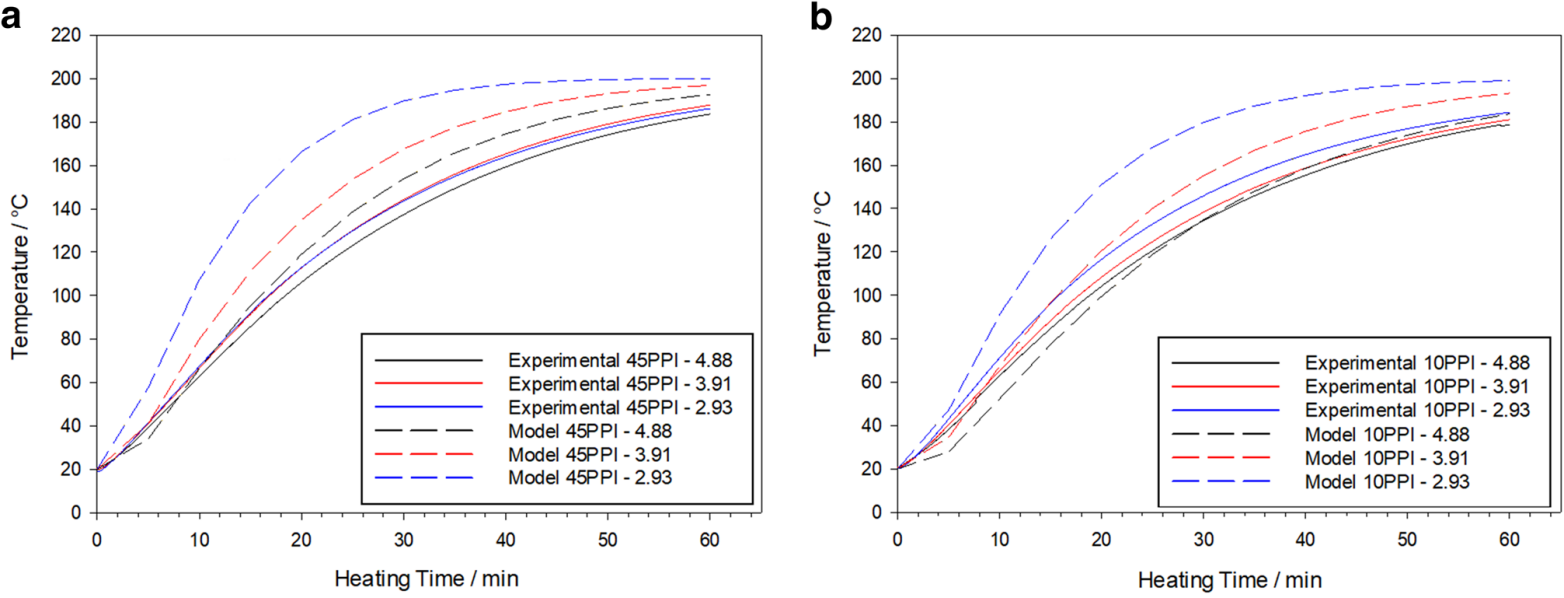
Thermocouple (solid line) and numerical simulations (dashed line) temperature verses heating time at foam centre for: (**a**) 45 PPI and (**b**) 10 PPI foam subjected to a volumetric compression ratio of 2.93 (blue line), 3.91 (red line) and 4.88 (black line). Softening temperature obtained through DSC (horizontal dashed line).

Figure 6 shows the mean measured temperature–time profiles at the centre of the 45 PPI and 10 PPI foams when subjected to VCRs of 4.88, 3.91 and 2.93, respectively. Although error bars are omitted for the sake of clarity, the variance within the 45 PPI and 10 PPI foam datasets is 2.2 °C, 4.2 °C, 1.5 °C and 3.6 °C, 1.4 °C, 2.1 °C for VCRs 4.88, 3.91 and 2.93, respectively.

VCR 3.91 and 2.93 exhibited a comparable heating rate until approximately 30 min at which point the 3.91 VCR heated at a faster rate. The 4.88 VCR exhibited the slowest heating rate. After 60 min all VCRs exhibited comparable internal temperatures between 184 to 188 °C. Unlike the 45 PPI foams, the 2.93 VCR exhibited the fastest heating rate, with the 4.88 VCR once again being the slowest. Again, all VCRs exhibited comparable final heating temperatures (between 179 and 185 °C), but at a lower value than their 45 PPI counterparts.

When compared to the experimental data (Fig. 6), the models show reasonable agreement for both foam types. Typically, the 45 PPI model predicts a greater heating rate for each VCR; however, a slower heating rate is shown for the 4.88 VCR up to 10 min. In contrast with the experimental data, the model predicts that the 2.93 VCR will exhibit the highest internal temperature after 60 min, with the 4.88 VCR still the lowest temperature. All VCRs are again predicted to have comparable internal temperatures after 60 min, but greater than their experimental counterparts, with values ranging between 193 to 200 °C.

Unlike the 45 PPI model, the 10 PPI model has a reasonably good agreement with the experimental data, giving the same order of highest to lowest final internal temperature. Similarly, for the 45 PPI foam model, the final predicted internal temperatures were greater than the experimental and ranged between 184 to 199 °C. Heating rate was again predicted to be faster than the experimental counterparts, although 4.88 and 3.91 VCRs were predicted to have a slower heating rate up to 30 and 10 min, respectively.

**Figure 7:**
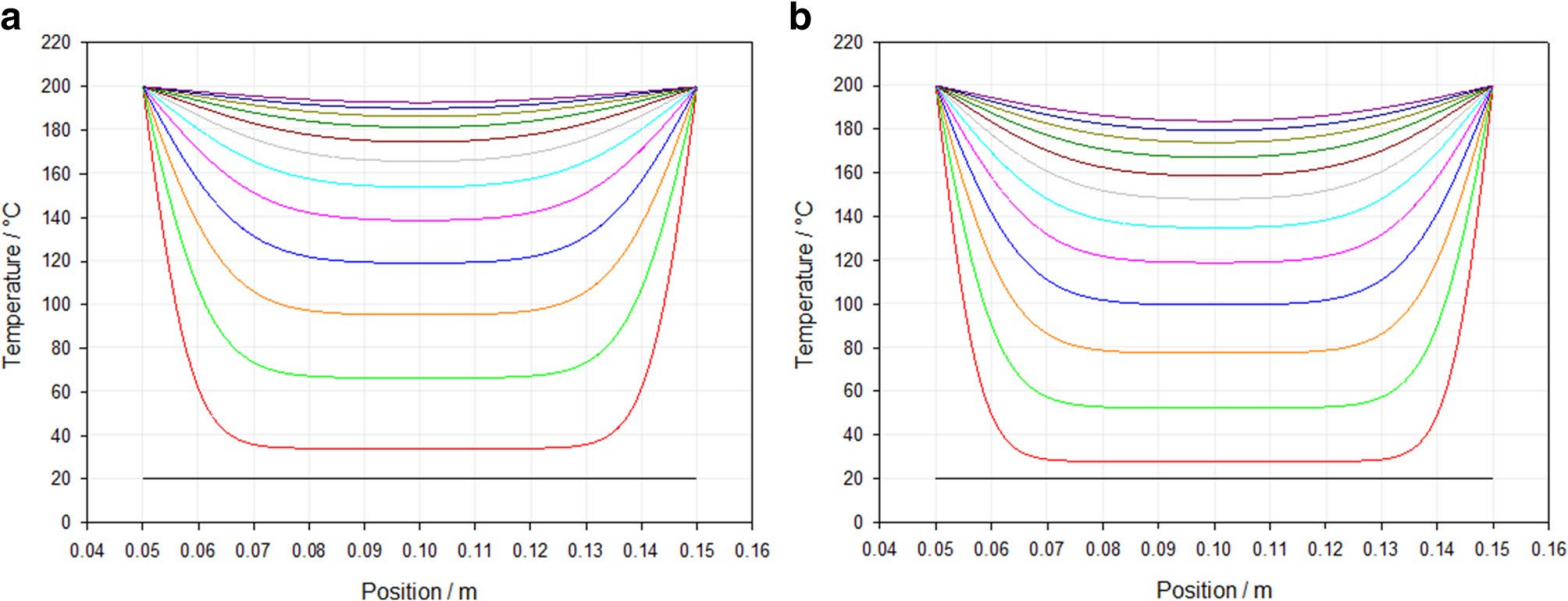
Predicted temperature distribution across the *x*-axis at the sample centre versus time for: (**a**) 45 PPI and (**b**) 10 PPI foams with a volumetric compression of 4.88.

When the study area was expanded to a cross-sectional area, a non-homogenous heating profile was indicated (Fig. 7). In all models, thermal equilibrium between the foam and heating source is predicted faster closest to the heating source and decreased with distance. As heating time increases the local temperature at any given point increases until thermal equilibrium is achieved.

**Figure 8:**
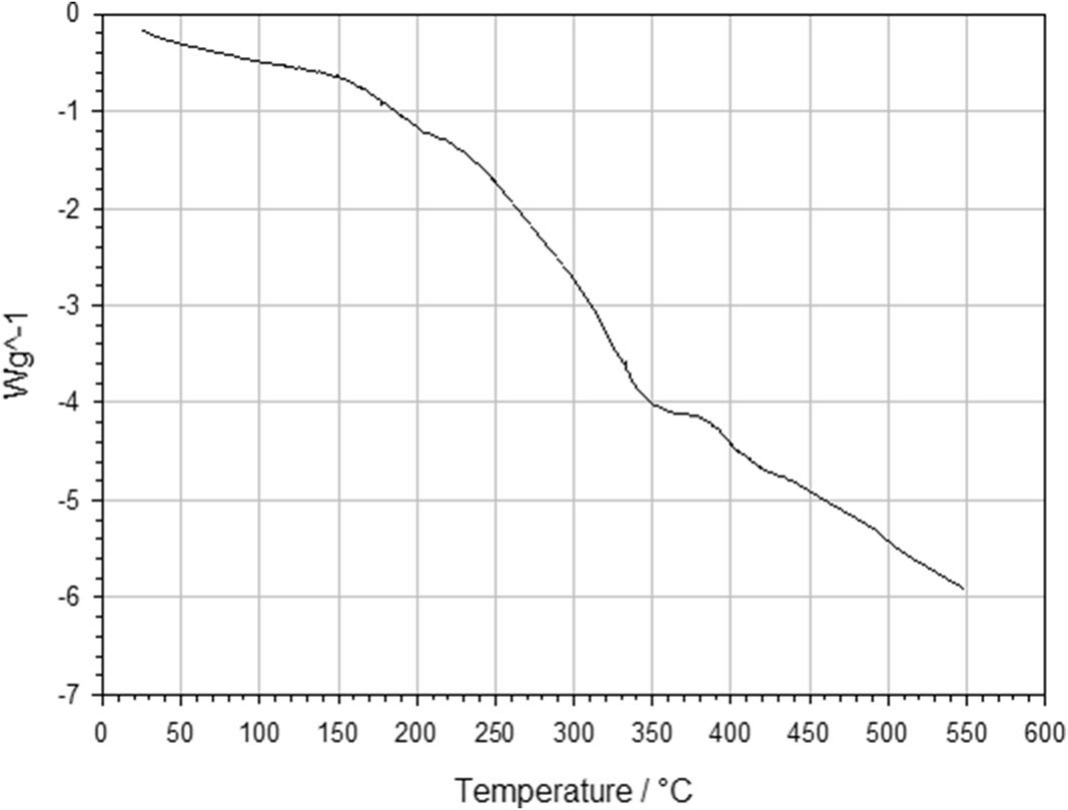
DSC analyse performed on the received PU foams, heated in the temperature range from 25 to 550 °C at a rate of 10 °C min^−1^, where a change in gradient occurs approximately at 150 °C, indicating a the start of softening.

Overall, 45 PPI foams were predicted to be heated faster than their 10 PPI counterparts, whilst a lower VCR predicted a faster heating rate which slowed as VCR increased. Assuming a softening temperature of 150 °C as indicated by the gradient change in DSC data (Fig. 8), the 2.93 VCR was predicted to achieve the fastest softening temperatures throughout the sample body, at a time of 17 and 20 min for the 45 PPI and 10 PPI foams, respectively. It was not until 36 min that all parameter combinations exceeded the softening temperature required to induce the auxetic structure.

### Volumetric compression ratio (VCR)

**Figure 9:**
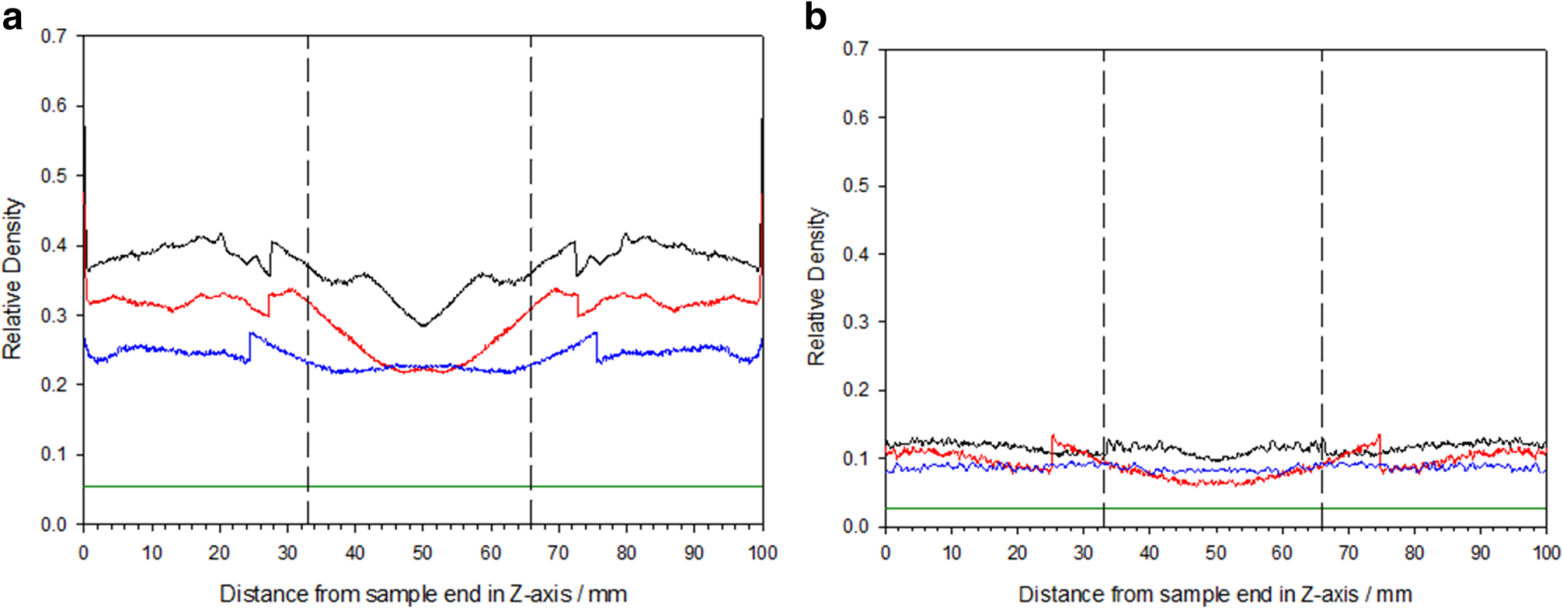
Relative density at a given location with respect to distance from sample edge using CT: (**a**) 45PPI and (**b**) 10 PPI foams for volumetric compression ratios of 2.93 (blue line), 3.91 (red line) and 4.88 (black line), where conventional foams are represented by a green line.

Figure 9 shows the relative density at a given location with respect to distance from sample end in the *z*-axis for each VCR and porosity combination. All plots exhibited distinct step changes, and are the direct result of the ISO50 standard, calculating different threshold values between sub-volumes. Currently the influence that the sub-volume offset has on the variation is unknown. Comparison with conventional foams (solid green line) indicates an increase in a sample’s overall relative density, by approximately the magnitude of the imposed VCR. The relative density change was shown to be heterogeneous, whereby density fluctuates depending on the distance from the sample edges. Typically, the highest relative density exists between 0 to 33 mm from the foam sample edges (either side of the vertical dashed lines). When distance from the end blocks exceeds approximately one-third of the volume, a decrease in relative density which converges to its lowest value at the sample centre.

Table 2 shows the mean density evaluated from the average relative density of the CT and calculated internal density. Converting relative density to true density by multiplying by the density of solid polyurethane, found that the average sample density was 447, 359 and 288 kg m^−3^ and 139, 112 and 104 kg m^−3^ for the 45 PPI and 10 PPI foams, respectively. Interestingly, when compared to the calculated density of the samples under compression (original volume divided by volume inside mould), it was found these values differed significantly.

**Table 2:** Mean density for each VCR and porosity combination from CT and calculated methods.

	Average relative density (CT)	Mean density (CT)/kg m^−3^	Calculated internal density/kg m^−3^
Auxetic 45 PPI—4.88	0.372 ± 0.032	447 ± 38	159 ± 3
Auxetic 45 PPI—3.91	0.299 ± 0.038	359 ± 46	130 ± 7
Auxetic 45 PPI—2.93	0.240 ± 0.014	288 ± 16	92 ± 2
Auxetic 10 PPI—4.88	0.115 ± 0.008	139 ± 9	203 ± 4
Auxetic 10 PPI—3.91	0.093 ± 0.017	112 ± 21	156 ± 4
Auxetic 10 PPI—2.93	0.087 ± 0.004	104 ± 5	113 ± 3

### Statistical analysis

A multiple regression analysis^55,56^ was performed in order to: (1) ascertain to what extent the variance in Poisson’s ratio was attributable to the parameters manipulated in the present experiment; (2) assess the unique contribution of each individual parameter in predicting the lowest Poisson’s ratio recorded in the present study; and (3) explore the interactions between experimental parameters. It is important to note that tri-axial compression fabrication lead to variation around the target VCRs of 4.88, 3.91 and 2.93, as captured in Table 3. Whilst it is also likely that slight variations may exist in the precision of manufacturer PPI and in the precision of heating time measurements, no such data is presently available for statistical comparison.

**Table 3:** Compression ratio variability.

Sample cases	Target VCR	Actual VCR (mean)	Actual VCR (min)	Actual VCR (max)	Actual VCR (SD)	Actual VCR (SE)
64	2.93	2.97	1.65	3.68	0.52	0.06
64	3.91	3.80	1.52	5.36	0.98	0.12
64	4.88	4.78	2.01	6.03	1.22	0.15

To account for VCR variation beyond the target ratios (i.e., 2.91, 3.91, 4.88) two multiple regression analyses complete with inspection of partial correlation coefficients were conducted. The first utilised target VCRs as taken from the literature and incorporated into the study, and the second utilised actual VCR measurements. Preliminary analyses were undertaken to ensure violations of parametric statistical assumptions (i.e., normality, linearity, multicollinearity, and homeoscedasticity) that could undermine statistical outputs could be ruled out.

**Figure 10:**
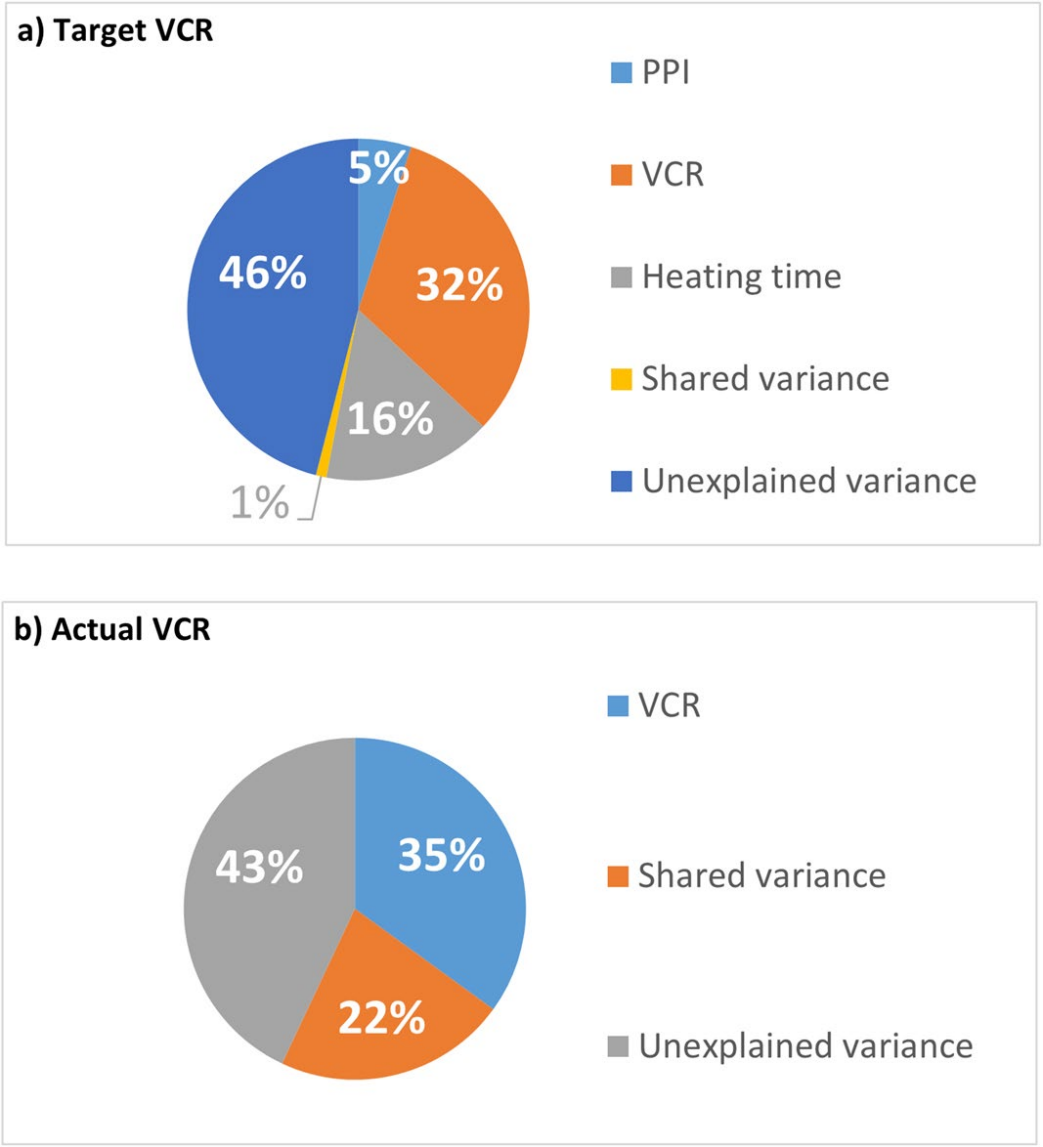
Predictive power of fabrication parameters based on: (**a**) Target VCR and (**b**) Actual VCR.

Multiple regression outputs indicate that PPI, VCR, and heating time collectively account for 54% of the variability in Poisson’s ratio (*r*^*2*^ (3, 188) = 0.54, *p* (*p* = the probability of false identification of experimental effects. Probability levels less than or equal to () 0.05 are deemed acceptable ^57^.) < 0.001) when examining the target VCR fabrication parameters and 57% of the variability in Poisson’s ratio (*r*^*2*^ (3, 188) = 0.57, *p* < 0.001) when considering actual VCR measurements. Examination of squared part correlation coefficients was used to determine the unique predictive power of the experimental fabrication parameters systematically varied in the present study (Fig. 10).

Both regression models indicate a marked portion of unexplained variance which can be attributed to experimental noise and further incidental fabrication parameters not captured in the present data. When discounting variance in Poisson’s ratio that cannot be attributed to the foam fabrication parameters presently manipulated experimentally (unexplained variance), VCR is consistently the largest predictor of (lowest) Poisson’s ratio, accounting for 32% of the unique variance in the target VCR model, and 35% of the unique variance in the actual VCR model (*p* < 0.001 in both instances). Further inspection of analytic outputs yields differing interpretations of the predictive power of PPI and heating time based upon VCR variability (target *vs*. actual VCR). When considering the target VCR model, both heating time and PPI explain a significant proportion of unique variance in NPR (16% and 5% respectively, *p* < 0.001 in both instances). However, neither of these parameters account for any unique variance in Poisson’s ratio in the actual VCR analysis (heating time *p* = 0.69, and PPI *p* = 0.45). Instead, the remaining variance in NPR as attributable to the present experimental parameters was shared variance representing the interaction between PPI and heating time. This outcome is likely as the result of contrasting actual (i.e., precise) VCR measurements to manufacturer PPI and pre-set experimental heating times (arbitrary categorical data) whose precise variability cannot be inferred or tested within the present dataset.

An alternate statistical approach, discriminant function analysis was afforded on the categorical foam fabrication parameters (i.e., the target parameters) incorporated into the present experimental design. This complementary analysis exploiting Chi-squared (χ^2^) goodness-of-fit statistics confirmed that variation in the designated parameters significantly predicted whether NPR was outputted (χ^2^(3) = 134.30, *p* < 0.001). It further confirmed that VCR was the greatest predictor of auxetic outcomes, followed by heating time, and finally PPI. Quantification of the probability (expressed as percentages (%)) that a set combination of manufacturing parameters had successfully produced an auxetic foam is displayed in Table 4.

**Table 4:** Estimated probability of successful production of auxetic foam, where success percentages greater than 90% are italic.

Heating time/mins	VCR 2.93		VCR 3.91		VCR 4.88	
	10 PPI (%)	45 PPI (%)	10 PPI (%)	45 PPI (%)	10 PPI (%)	45 PPI (%)
25	0.09	1.16	0.96	11.47	9.64	58.81
30	0.19	2.45	2.02	21.65	18.53	75.28
35	0.40	5.08	4.21	37.09	32.67	86.66
40	0.85	10.24	8.59	55.71	50.87	*93.27*
45	1.79	19.58	16.70	72.85	68.83	*96.73*
50	3.74	34.19	29.92	85.13	82.49	*98.44*
55	7.65	52.57	47.70	*92.43*	*90.95*	*99.26*
60	15.01	70.27	66.06	*96.30*	*95.55*	*99.65*

### Discussion

Auxetic foams were produced over a range of fabrication parameter combinations: including VCR, PPI and heating time, with each parameter playing a significant role in the thermo-mechanical process. Volumetric compression ratio was shown to be the primary parameter in determining the auxeticity of the foam sample, while heating times determined the stability of the sample. These results were supported statistically, with multiple regression demonstrating that systematic variation in PPI, VCR and heating time collectively account for the majority of variance in Poisson’s ratio (54–60%) and discriminant function analysis pinpointing the levels of manufacturing parameters with the greater likelihood of producing auxetic foams. Regarding individual manufacturing parameters, statistical analyses consistently identified VCR as the strongest predictor of whether NPR was achieved. Regression analysis returned mixed evidence as to the predictive power of PPI and heating time but indicated that heating time was more important than PPI. Discriminant function analysis confirmed this conclusion.

Discriminant function analysis was exploited to assign percentage probabilities to the success of the differing manufacturing parameter combinations in producing auxetic outputs. Representing a novel contribution to the scientific field, further discriminant analysis results indicate that of the 48 unique combinations of foam manufacturing parameters studied presently, only nine (19%) had a greater than 90% chance of producing auxetic foams. Discussion now turns to the behaviour of individual manufacturing parameters.

As VCR reduced, samples exhibited lower NPRs until returning to a positive Poisson’s ratio, behaviour in agreement with^30^, and was the direct result of cell rib deformation. At lower VCRs, only minimal cell rib deformation occurs due to the restrictions of the internal cell space. With increasing compression, the ribs continue to deform in a random concave manner to occupy the limited space^12^. Should the VCR become too high, all internal cell space is likely to become occupied with deformed cell ribs, and no further compression may be undertaken; thus, corroborating the recommended ratio between 2 and 5 reported within the open literature^24,37^.

When compared to the wider literature^3,13,16,58–61^, it was found that the NPR values reported within were typically comparable. The most prominent example can be found in^16^ when cylindrical 45 PPI foams of substantially smaller volume (up to 2.7 times) were subjected to VCRs ranging from 5.0 to 19.1 resulted in NPRs between − 0.63 to − 0.09. These values were almost identical to those reported for the same PPI herein (− 0.10 to − 0.67), while achieved using heating temperatures and times much lower than those reported here (135–150 °C and 12–15 min). While a distinct breakdown of the final NPR for each VCR in was not provided, the authors come to the same statistical conclusion that VCR is the most important factor between all the manufacturing parameters, even though the fabrication parameters between their study and this one wildly vary. The same behaviour can be further inferred from^13,59,61^ where further variations in fabrication parameters (including specimen geometry, PPI, heating temperature and time) find that as VCR reduces so does the NPR.

This behaviour was further confirmed using computed tomography and supported by^19,62^, where an increase in relative density was shown at higher VCRs. Relative density was shown to be heterogeneous throughout the sample body and be highest at the end thirds of the sample. This is potentially attributed to localised collapse (crumple zones) of the cell ribs closest to the foam surface in contact with the end blocks, as previously reported^63,64^. Interestingly, this behaviour differs to that reported by Elliot et al.^63^, where the highest relative density exists at the sample centre. Although unclear why these differences exist, it is likely the product of the different compression methods utilised between studies.

When relative density was converted to a regular density, the values were higher when compared to the calculated density of the samples under compression. Confidence was given to the calculated values, as comparable densities were measured post fabrication. It is suspected that discrepancy is the by-product of the tomographic analysis method, especially the binarising phase. Prior to binarsing, should any artefacts (e.g., mould, foam creases, etc.) be present, they have the potential to be categorised as material, and thus increase the relative density. The ISO50 standard also contributes to this behaviour, as it determines the threshold of what is considered material.

A decrease in heating time was also shown to reduce the negative Poisson’s ratio. Unlike VCR, which compresses the cells into an auxetic concave arrangement, heating time affects sample stability. Heating time by itself however can be considered a misleading parameter to judge auxetic fabrication, as it often relates to a specific experimental study, and not auxetic manufacturing as a whole. This is best shown by the large variance in fabrication parameters reported in literature which have produced successful auxetic samples. For example, if the parameters reported in^16^ were used with the sample geometries implemented in this study, auxetic samples are unlikely to be produced as the softening temperature is unlikely to be exceeded. To overcome this issue, the relationships between sample material, volumetric compression, heating time, and mould geometry need to be developed. Such a timely undertaking, however, may prove challenging when more complex geometries are considered.

Overall, 45 PPI foams were more effective at auxetic conversion (57%) compared to the 10 PPI which only had a conversion rate of only 26%, and typically agree with Wang et al.^24^, who report smaller cell sized foams require shorter processing times than their larger counterparts. It is suggested that this behaviour could be due to either the surface tension effects within smaller cells or to a possible difference in the chemistry of the material. Statistically, variation in PPI was found to be the least powerful predictor of Poisson’s ratio out of all parameters investigated presently. This may in part be due to having only two levels of PPI (10 and 45). Further experimentation, investigating a wider array of PPI levels would be required to determine the predictive power of porosity more conclusively on Poisson’s ratio.

In the rare instances anisotropic NPRs were measured, this behaviour is suspected to be a product of the nonhomogeneous structure of the base foam. For all samples tested, the foam rise direction was in the lateral axis. Since pores in the rise direction are often elongated in shape, when subjected to a VCR, these pores are likely to experience a greater compression in the lateral direction than the axial due to about the elongated cell walls having a reduce resistance to bulking (due to their length/width ratio). When the tensile axial load is then applied, a greater expansion in the lateral direction will then be measured. Should the cells within the measured lateral plane experience high levels of local compression, an anisotropic NPR may occur. This notion is supported by^65,66^ where local NPRs have been measured using Digital Volume Correlation. Experimental error such irregular loading during testing, in proper fixture of the sample, inaccuracies in dimensional change measurement or the presence of localised creasing could also play a role.

At higher heating times, samples are more stable as they experience greater thermal exposure, which in turn causes the compressed foam to heat to higher temperatures. Should heating times be sufficient in duration, the foam will exceed the softening temperature^12^, and thus fix the auxetic structure. For insufficient times the foam will fail to reach the softening temperature and not fix the auxetic structure. This behaviour is evidenced by both thermocouple and computation data, where central, samples exceed the softening temperature at times as low as 17 min, respectively. Although it is inconclusive why the models over predict the heating behaviour, it is suspected that the principle factor is the homogeneity of the foam material simulated within COMSOL. Unlike real foams, which comprised of a randomly distributed foam cell network, it is suspected that COMSOL homogenises the porous media into a single medium^46^.

When the model was expanded to consider full body heating, a non-uniform temperature distribution is predicted throughout the samples and suggests that the outer regions of the samples achieve softening temperature faster than the centre. It is conceivable that this non-uniform temperature distribution can explain the transition period reported in this study. During the transition period it is likely that the foam experiences three distinct regions; auxetic, weakened auxetic and conventional. Cells with a weakened auxetic structure are likely to not have achieved the softening temperature but be close. As such, their auxetic structure is unstable, as the cell stress relief has yet to occur. As such when subjected to a mechanical tensile stimuli, the stresses within the cells overcome the auxetic structure and permanently return to an unstressed conventional cell structure. Should enough weakened auxetic cells return to a conventional form, and the ratio of auxetic to conventional cells increases in favour of the conventional cells, a positive Poisson’s ratio is likely to occur. It is expected that the ratio of non-auxetic to auxetic cells influences auxetic behaviour and NPR exhibited by a sample.

Non-homogenous heating could explain the instability changes observed in literature^3,17^. Within these samples, if the softening temperature region is greater than the non-softening region, the increased stiffness of the auxetic region should be sufficient to resist the volumetric change. However, if the alternative scenario occurs, then it is likely that the compressed cells will overcome the stiff auxetic cells, thus inducing volume change over time.

Before drawing conclusions, it is important to address the levels of unexplained variance indicated within the multiple regression analysis—46% and 43% variance in the target and actual VCR comparisons respectively (as displayed in Fig. 10). Whilst a portion of the variance will be as a consequence of experimental measurement noise, there is also the possibility that further fabrication factors might be at play. It was not possible to capture data concerning the following factors:

- Variation in ambient room temperature (21–23 °C) effecting sample cooling times;
- Relative humidity;
- Sample creasing;
- Oven time/temperature fluctuations (minute variations in the time taken for the oven to reach the intended temperature once the fabrication moulds were inserted into the oven. Also, variation in oven time due to the time taken to load and unload the fabrication moulds).

These factors represent limitations on the present experimental design. Whilst it may prove difficult to successfully control for all of these factors, future manufacturing studies would benefit from considering how best to capture data that can be explored in future statistical investigations.

## Conclusions

Auxetic foams were produced over a range of fabrication parameters combinations including VCR, PPI and heating time, with each parameter playing a significant role in the thermo-mechanical process, as demonstrated both computationally and statistically. Overall, 45 PPI foams were more effective at auxetic conversion (57%) compared to the 10 PPI which only had a conversion rate of only 26%. Volumetric compression ratio was shown to be the primary parameter in determining the sample auxeticity, while heating time determined the stability of the sample. It is recommended that those wishing to maximise the efficiency of auxetic foam production should utilise samples with a VCR of 4.88, a porosity of 45 PPI, and a heating time of between 40–60 min.
