# Author Correction: Experimental and computation assessment of thermomechanical effects during auxetic foam fabrication

**DOI:** 10.1038/s41598-021-88211-w

**Published:** 2021-04-13

**Authors:** Richard Critchley, Victoria Smy, Ilaria Corni, Julian A. Wharton, Frank C. Walsh, Robert J. K. Wood, Keith R. Stokes

**Affiliations:** 1 Survivability and Advanced Materials, Centre for Defence Engineering, Cranfeld University, Defence Academy of the United Kingdom, Shrivenham, SN6 8LA UK; 2 National Centre of Advanced Tribology at Southampton (nCATS), Mechanical Engineering, Faculty of Engineering and Physical Sciences, University of Southampton, Highfeld, Southampton, SO17 1BJ UK; 3 Applied Cognitive Science Group, Centre for Electronic Warfare, Information and Cyber, Cranfeld University, Defence Academy of the United Kingdom, Shrivenham, SN6 8LA UK; 4 nC2, Enterprise Consultancy Unit, University of Southampton, Southampton, SO17 1BJ UK

Correction to: *Scientific Reports* 10.1038/s41598-020-75298-w, published online 27 October 2020

This Article contains an error. A Data Availability section was originally not included—it should appear as below:

**Data Availability**

Data underlying this study can be accessed through the Cranfield University repository at 10.17862/cranfield.rd.13186745.v1.

